# Alcohol’s Effects on the Risk for Coronary Heart Disease

**Published:** 2001

**Authors:** Kenneth J. Mukamal, Eric B. Rimm

**Affiliations:** Kenneth J. Mukamal, M.D., M.P.H., M.A., is an instructor in medicine at Harvard Medical School, and associate physician at Beth Israel Deaconess Medical Center, Boston, Massachusetts. Eric B. Rimm, Sc.D., is an associate professor of nutrition and epidemiology at Harvard School of Public Health, and assistant professor of medicine at Harvard Medical School, Boston, Massachusetts

**Keywords:** heart disorder, risk factors, beneficial vs. adverse drug effect, moderate alcohol and other drug (AOD) use, AOD abstinence, lifestyle, high density lipoprotein, platelet aggregation, blood pressure, endothelial cell, genetics and heredity, alcohol dehydrogenases, pathologic process, meta-analysis

## Abstract

Several studies have indicated that moderate drinkers have a lower risk of both nonfatal myocardial infarction and fatal heart disease than do abstainers. To determine whether alcohol truly prevents coronary heart disease or whether other factors may contribute to this observed relationship, researchers conducted a systematic literature review and a combined analysis (i.e., meta-analysis) of 42 published studies. This analysis found that consumption of up to two drinks per day can promote changes in the levels of molecules that reduce the risk of heart disease while also increasing the levels of certain molecules that promote heart disease. Alcohol also may affect the risk of heart disease by acting on other various other molecules involved in a variety of physiological processes related to heart disease. Finally, the relationship between alcohol consumption and heart disease may be modulated by genetic factors.

Since the early part of the 20th century, clinicians have noted that coronary heart disease appears to occur less commonly among people who consume alcohol than among abstainers. Over the last 30 years, formal scientific inquiry has confirmed this observation. Such analyses included studies that compared alcohol use between people with and without confirmed coronary disease (i.e., case-control studies) as well as studies that followed healthy drinkers and abstainers over time to determine their risk of coronary disease (i.e., prospective cohort studies). Both types of studies found that people who consumed alcohol in moderation had lower rates of coronary heart disease compared with abstainers. For example, in a prospective study of 51,529 healthy men, Rimm and colleagues (1991) found that men who consumed 5.1–30 grams of alcohol (about 0.3–2 standard drinks^1^) per day had a 29 percent lower risk of suffering either nonfatal myocardial infarction or fatal heart disease than did abstainers. Similarly, the drinkers had a 16 percent lower risk of undergoing bypass surgery or angioplasty compared with abstainers. These findings were confirmed in a review of over 50 epidemiological studies, which concluded that compared to total abstinence, consumption of one drink every 1 to 2 days is associated with a 17 percent lower risk of nonfatal myocardial infarction (Maclure 1993).

This article reviews the evidence that it is indeed the consumption of alcoholic beverages rather than other unrelated factors that reduces the risk of coronary heart disease. It also presents a recent approach to determine the relationship between alcohol consumption and coronary heart disease. This approach explores alcohol’s influence on known risk factors for coronary disease as well as other pathways through which alcohol may affect the risk for heart disease. Finally, the article investigates the role of genetic factors in modifying the relationship between alcohol consumption and the risk of coronary heart disease.

## Does Alcohol Truly Prevent Coronary Heart Disease?

Although the evidence of a lower risk of coronary heart disease among moderate drinkers is substantial and consistent, controversy remains about whether the relationship is truly causal—that is, whether moderate alcohol consumption really prevents coronary heart disease. For example, some investigators have argued that abstainers are an inappropriate control population because at least some of these people may abstain because of illness or former alcohol abuse. Furthermore, other dietary, lifestyle, and developmental factors may differ between abstainers and drinkers. Both of these concerns warrant closer scrutiny.

The first of these concerns, also called the “sick quitter” hypothesis, was proposed by Shaper and colleagues (1988) in the United Kingdom. It states that the pool of abstainers includes many former drinkers who quit drinking because of illness or because alcohol interacts with prescription drugs they are taking. Obviously, comparisons of healthy drinkers with abstainers who take prescription drugs or who have underlying illnesses that raise one’s risk for heart disease will produce a biased result in favor of the alcohol-consuming subjects.

Similarly, alcoholic patients in recovery rarely return to moderate, or social, drinking. Thus, people who have alcoholism, whether still active or in remission, will tend toward the extremes of alcohol consumption (i.e., abstention or heavy drinking). People with active alcoholism, however, tend to be under-represented in large, prospective studies of heart disease. As a result, comparison of drinkers (who underrepresent active alcohol abusers) to abstainers (who include recovering alcoholics) may produce misleading results if heavy alcohol consumption contributes to a higher risk of heart disease. A similar bias may occur if, in self-report surveys, people who consume alcohol in excess deliberately describe themselves as abstainers because of the social stigma attached to alcohol abuse.

Other fundamental differences also exist between abstainers and drinkers. For example, abstainers tend to come from less welcoming childhood environments^2^ and to report poorer health than do drinkers, even in early adulthood (Vaillant 1995). Moreover, many abstainers have chosen to forsake alcohol intake because of adverse experiences with alcoholic family members. Such differences, which might influence a person’s underlying risk of heart disease, are difficult to account for with standard epidemiological or statistical methods.

Finally, the level of alcohol consumption is a marker for several lifestyle factors that strongly influence health (Fillmore et al. 1998). Moderate drinkers tend to be younger, leaner, more physical active, of higher socioeconomic status, and more likely to be married compared with people who abstain or drink rarely. All of these factors have been shown to influence one’s risk of coronary heart disease.

### Approaches for Accounting for Potential Confounding Factors

Researchers have sought to address these concerns in several ways. Some epidemiological studies have separated former drinkers from long-term abstainers to address the sick quitter hypothesis. For example, in an analysis of 87,526 women, the risk of coronary heart disease was only 10 percent higher among former drinkers than among long-term abstainers (Stampfer et al. 1988). Furthermore, the exclusion of former drinkers among the abstainers did not alter the 40 percent lower risk of coronary heart disease among women who drank 5.0–14.9 grams of alcohol (about 0.3–1 standard drinks) daily. Moreover, Rimm and colleagues (1991) found comparable risks of coronary heart disease among abstainers and light drinkers (i.e., people who consumed less than 5.0 grams of alcohol, or 0.3 standard drinks, daily) in their study of 51,529 healthy men, suggesting that abstainers are not an inappropriate reference group. Other studies have excluded participants who developed coronary heart disease or died during the first few years of followup, as a means of excluding unidentified “sick” subjects, with similar results (Fuchs et al. 1995). Taken together these findings indicate that the presence of sick quitters or former alcoholics among the abstainers is not responsible for the apparent benefits of alcohol consumption on the risk of coronary heart disease.

Researchers also have sought to separate the confounding influence of dietary, lifestyle, and socioeconomic factors from the role of alcohol consumption itself. In the separate prospective studies of men and women noted earlier (Rimm et al. 1991; Stampfer et al. 1988), researchers controlled for the participants’ body-mass index (a measure of obesity) and dietary intake of cholesterol, saturated fat, and polyunsaturated fat. These analyses confirmed that diet alone is unlikely to have caused the apparent effect of alcohol consumption on heart disease. Studies that have controlled for the participants’ social integration, social class, physical activity, or occupation have reported similar results (Murray et al. 1999).

Another area of controversy remains the role of the type of beverage a drinker consumes preferentially. The French paradox—the observation that the rate of coronary heart disease in France is relatively low despite high rates of saturated fat intake and cigarette smoking—has led to the belief that red wine is particularly beneficial for health. This specific effect has been suggested to result from the antioxidant^3^ properties of some components of red wine rather than its alcohol content. However, observational studies have not consistently shown a difference in the risk of heart disease between wine drinkers and consumers of other alcoholic beverages (Rimm et al. 1996).

In summary, all of this evidence implicates alcohol consumption rather than lifestyle factors (including those that correlate with the consumption of specific beverage types) as the primary factor in the lower rates of cardiovascular disease found among moderate drinkers.

## Approaches to Defining the Causal Role of Alcohol

Although investigators have used a variety of approaches to address the aforementioned concerns, the most definitive way to determine whether alcohol consumption itself prevents coronary heart disease would be to conduct a randomized controlled trial. In a randomized trial, investigators randomly assign study participants to receive either the treatment of interest (e.g., a certain medication or alcohol) or a control treatment (e.g., an inactive substance called a placebo). Because the participants are randomly assigned to the active or control treatment and all participants are equally likely to receive a given treatment, the design of a randomized trial minimizes the effects of other variables. For an agent such as alcohol, which is influenced by, and in turn influences, so many other factors, the advantages of a randomized study design are particularly useful.

Unfortunately, however, even the randomized trial is not a perfect tool for determining the relationship between alcohol consumption and coronary heart disease. No long-term trial of alcohol administration has ever been performed, nor is one likely in the near future. Such a trial would face substantial hurdles, including the following:

- High costs
- An inability to prevent participants from knowing whether they receive alcohol (i.e., to blind participants to alcohol exposure),
- The need to find large numbers of people who are not prevented from using alcohol for medical reasons and who are willing to forgo or continue alcohol use for long periods of time, and
- The possibility that some participants instructed to consume alcohol would eventually misuse it or even become alcohol dependent.

This latter ethical concern—which also includes the possibility that a study participant assigned to the alcohol-consuming group might injure him-or herself or another person (e.g., in an alcohol-related car crash)—raises serious doubts whether a long-term randomized trial of alcohol use is at all possible (Tucker and Vuchinich 2000).

In contrast, researchers have conducted many randomized short-term trials of alcohol administration and its consequences. These trials avoid the pitfalls of observational studies (e.g., the influences of factors that cannot easily be measured) and the concerns associated with long-term trials. The interpretability of such short-term studies, however, often is limited. For example, short-term trials of alcohol use are necessarily limited to studying physiological measures (e.g., changes in blood cholesterol levels) rather than clinical endpoints (e.g., the development of heart disease). The relevance of such physiological measures to clinical heart disease, however, is often uncertain. Furthermore, because they often include small numbers of subjects, these trials produce relatively imprecise results.

## A Meta-Analysis of Existing Trials

To address the limitations of existing short-term trials of alcohol administration mentioned above and thus make those trials more meaningful, Rimm and colleagues (1999) analyzed the relationship between alcohol consumption and coronary heart disease using two complementary approaches. First, the investigators performed a meta-analysis—a systematic review of the existing literature that combines separate studies to yield a single, quantitative summary score. In a meta-analysis, the studies that are collected during the literature review are scrutinized carefully, using explicit and predetermined inclusion criteria. The investigators then determine an “average” effect across the pooled studies, giving the greatest weight to the largest studies. In their meta-analysis, Rimm and colleagues (1999) determined the overall effect of alcohol consumption on several biological markers of cardiovascular risk based on 42 published trials. All of these trials offered the advantage of a randomized design but were limited by their small size.

Second, the researchers assessed how changes in specific risk factors affected the risk of coronary heart disease in published prospective studies of risk. Finally, the investigators combined these two approaches to gain a sense of the extent to which alcohol-related changes in coronary risk factors in randomized studies would be expected to influence the risk of coronary heart disease. Consistent results between this meta-analysis and existing observational studies would provide further evidence that alcohol itself can prevent coronary heart disease. To better understand the risk factors studied in the meta-analysis, the sidebar (p. 258) provides a review of the physiological processes contributing to an acute myocardial infarction.

**Precursors To Coronary Heart Disease**

An acute myocardial infarction or heart attack occurs when one of the blood vessels that supplies the heart muscle with oxygen and nutrients becomes blocked. This typically occurs when a blood clot becomes lodged in an artery that is already partially blocked by cholesterol deposits and other substances. As a result, the part of the heart muscle that is supplied by the blocked blood vessel can no longer function normally, which compromises the heart’s ability to pump blood effectively.

Three intertwined processes govern the development of an acute myocardial infarction: atherosclerosis, inflammation, and thrombosis. The term “atherosclerosis” refers to the deposit of increasing amounts of cholesterol in the cells of the blood vessel wall (i.e., endothelial lining). These deposits eventually form an atherosclerotic plaque that at least partly blocks the blood flow through that vessel. The process of cholesterol deposition is dynamic, however, with cholesterol simultaneously accumulating in and being removed from the vessel wall. These processes occur at different rates and depend on the amount and type of cholesterol primarily found in the blood. For example, high blood cholesterol levels, particularly in the form of a type of cholesterol called low-density lipoprotein cholesterol (LDL-C; “bad” cholesterol), promote cholesterol deposition. Conversely, a type of cholesterol called high-density lipoprotein cholesterol (HDL-C; “good” cholesterol) promotes a “reverse transport” that removes cholesterol from fatty, or lipid, particles and takes it back to the liver, where it can be removed from the body.

The body considers an atherosclerotic plaque a foreign body within the blood vessel wall. As a result, as the atherosclerotic process progresses, inflammatory cells invade the plaque in an effort to attack this foreign body. These inflammatory cells may promote further lipid deposition, damage the endothelial lining, and destabilize the atherosclerotic plaque, predisposing it to rupture. When such a plaque ruptures, various types of molecules that promote blood clot formation (i.e., coagulation) come into contact with the blood. This process, in turn, promotes thrombosis—the formation of a blood clot, or thrombus—over the bulky plaque, which may complete the blockage of the blood vessel.

Coagulation, however, also is a highly complex and dynamic process that involves numerous molecules. Thus, some proteins that circulate in the blood (e.g., fibrinogen and factor VII) tend to promote thrombosis, as part of the clotting cascade. Conversely, other molecules that act only locally (e.g., tissue-type plasminogen activator [t-PA]) can help dissolve newly formed thrombi, a process called fibrinolysis.

Other cells and molecules also can be drawn into the complex process of blood clot formation and dissolution. For example, fibrinogen and other coagulation-promoting molecules activate platelets—a type of blood cell that helps to seal off injured blood vessels—and encourage platelet aggregation at the site of the plaque rupture. Again, this aggregation can enhance the blockage of the affected blood vessel. Meanwhile, other substances, such as plasminogen-activator-inhibitor 1 (PAI-1), temper the fibrinolytic process.

In most cases, the formation of a new thrombus when an atherosclerotic plaque ruptures is the final step in the blockage of a coronary vessel and the initiation of a myocardial infarction. Thus, the three processes of atherosclerosis, inflammation, and thrombosis interact to produce clinical coronary heart disease.

—Kenneth J. Mukamal and Eric B. Rimm

### Alcohol’s Effects on Coronary Risk Factors

As mentioned previously, the findings of observational studies had suggested that alcohol consumption was inversely related to myocardial infarction. These findings were confirmed by the meta-analysis of short-term trials of alcohol administration, which indicated that alcohol consumption has important effects on factors involved in atherosclerosis, inflammation, and thrombosis. The most important of these effects is on high-density lipoprotein cholesterol (HDL-C, “good” cholesterol) levels. Thus, in their meta-analysis, Rimm and colleagues (1999) estimated that consumption of 30 grams of alcohol, or approximately two standard drinks, per day increases HDL-C levels by 4.0 milligrams per deciliter (mg/dL). This increase in HDL-C levels is greater than that produced by gemfibrozil, a medication used to treat people with low HDL-C levels and translates into a 16.8 percent decrease in the risk of coronary heart disease (Stampfer et al. 1991).

At the same time, however, alcohol consumption raises the levels of another type of fat in the blood—the triglycerides, which are associated with an increased risk of coronary heart disease. In the randomized trials included in the meta-analysis, consumption of 30 grams of alcohol raised triglyceride levels by an estimated 5.7 percent, which translates into a 4.6 percent increase in coronary heart disease (Stampfer et al. 1996). Thus, alcohol has a mixed effect on coronary risk factors by increasing both HDL-C and triglyceride levels. The balance of these effects, however, appears to favor prevention of coronary heart disease.

The meta-analysis also detected important effects of alcohol consumption on blood-clotting (i.e., coagulatory) factors. The best studied of these factors is fibrinogen, which is converted to fibrin during blood clot formation. The randomized short-term trials of alcohol administration included in the meta-analysis indicated that consumption of 30 grams of alcohol lowered fibrinogen levels by an estimated 7.5 mg/dL. This degree of reduction in fibrinogen concentration would be expected to reduce the risk of heart disease by 12.5 percent (Meade et al. 1986).

Taken together, the estimated changes in HDL-C, triglyceride, and fibrinogen levels induced by consumption of 30 grams of alcohol appear to result in a 24.7 percent reduction in the risk of coronary heart disease. Thus, the results of the randomized trials included in that meta-analysis support the hypothesis that alcohol indeed is the cause of the lower rates of coronary heart disease found among moderate drinkers, although additional research is needed to prove this assumption.

### Other Potential Mechanisms of Alcohol’s Effects

Alcohol consumption may also affect the risk of coronary heart disease by acting on other proteins involved in blood clot formation and fibrinolysis, as well as on platelet aggregation, blood pressure, and inflammation. For example, the meta-analysis by Rimm and colleagues (1999) found that consumption of 30 grams of alcohol raises the levels of the fibrinolytic protein tissue-type plasminogen activator (t-PA) by approximately 20 percent. Such an increase in t-PA levels might be expected to lower the risk of coronary heart disease; however, observational studies found the opposite effect (i.e., an increased risk of heart disease). One explanation for this observation might be that higher t-PA concentrations generally reflect more extensive underlying vascular disease. Alternatively, much of the measured t-PA in the blood may be bound to its inhibitor, plasminogen activator inhibitor-1, and therefore be inactive.

Furthermore, the meta-analysis found that intake of 30 grams of alcohol is associated with a 0.70 mg/dL decrease in the levels of a molecule called Lp (a) lipoprotein, which is a lipid particle that may influence fibrinolytic activity (Rimm et al. 1999). The relationship between Lp (a) lipoprotein and the risk of vascular disease has been inconsistent, however.

Alcohol also appears to inhibit platelet aggregation (Rubin 1999). This observation was confirmed in the randomized experimental studies included in the meta-analysis, which used a variety of assays. These studies found generally consistent evidence that alcohol consumption prevents platelet aggregation. Although the relevance of these findings for the risk for coronary heart disease is less clear than with confirmed coronary risk factors, such as HDL-C or fibrinogen, alcohol’s effects on platelet activity could represent an important mechanism through which alcohol could prevent cardiovascular disease.

Alcohol also appears to have important effects on other cardiovascular risk factors. Among the most controversial of these risk factors is blood pressure. Both epidemiological evidence and clinical trials confirm that heavy drinking (i.e., three or more standard drinks per day) raises blood pressure, both among people with and without elevated blood pressure (i.e., hypertension) prior to alcohol consumption (Keil et al. 1998). However, the effects of smaller amounts of alcohol on blood pressure have not been widely tested in randomized trials, and observational studies do not support a substantial effect on blood pressure from moderate drinking.

Alcohol intake may also affect the inflammation associated with atherosclerotic plaques and the function of the cells that line the blood vessels (i.e., endothelial cells). Observational studies indicate that moderate drinkers have lower levels of markers of inflammation. These markers include a molecule called C-reactive protein that is produced during inflammatory states and which has been linked to increased risk of coronary heart disease (Ridker et al. 1998). Conversely observational studies also indicate that moderate drinkers have higher levels of homocysteine, a substance derived from breakdown of the amino acid methionine that may increase the risk of blood clots. Only modest experimental data exist, however, to confirm either relationship (Imhof et al. 2001; Jacques et al. 2001).

Small studies have found mixed results of the effect of alcohol consumption on the function of the endothelial lining of the blood vessels. Laboratory experiments have suggested that regular alcohol consumption might increase the endothelial cells’ production of and responsiveness to nitric oxide, a small molecule made in blood vessel walls that helps to relax constricted blood vessels and thereby improve blood flow to organs such as the heart. If these findings can be confirmed, they could suggest another mechanism through which alcohol consumption may prevent myocardial infarction.

## The Role of Genetic Factors in the Association of Alcohol and Heart Disease

One unresolved question is whether the relationship of alcohol consumption to heart disease is consistent throughout the general population or differs among certain subgroups (e.g., men and women). For example, because men and women differ in how they metabolize alcohol and in their underlying risk of cardiovascular disease, they may also differ in how alcohol consumption relates to their risk of heart disease. Such variability is difficult to assess in randomized trials of alcohol consumption, which have been too small to allow subgroup comparisons. Observational studies, however, provide some intriguing answers to this question. For example, the studies of healthy men (Rimm et al. 1991) and women (Stampfer et al. 1988) mentioned previously suggest that moderate drinking is associated with lower risk of heart disease in both sexes, despite the differences in alcohol metabolism and risk of cardiovascular disease. These studies also demonstrate, however, that the level of alcohol consumption associated with the lowest risk of heart disease is lower among women than among men, consistent with public health recommendations that advise consumption of no more than two drinks per day for men and no more than one drink per day for nonpregnant women.

Genetic factors may also modify the relationship between moderate drinking and coronary heart disease in interesting ways. For example, the initial breakdown of the alcohol contained in alcoholic beverages—chemically referred to as ethanol—is mediated by an enzyme called alcohol dehydrogenase (ADH). Three different versions of ADH exist— ADH1, ADH2, and ADH3. Of these, ADH3 has two common genetic variants, or alleles, that break down ethanol at different speeds (i.e., fast and slow). Each person carries two copies of the ADH3 gene, one inherited from the father and one inherited from the mother. Accordingly, a person can carry either two fast alleles, two slow alleles, or one fast and one slow allele of the ADH3 gene.

A recent study of 396 men with myocardial infarction and 770 control men studied the relationship between these ADH3 alleles and the risk of heart disease (Hines et al. 2001). The study found that compared with men who carried two copies of the fast allele and drank less than once per week, men who carried two copies of the fast allele and drank daily had a 38 percent lower risk of myocardial infarction. In contrast, daily drinkers who had two copies of the slow allele had an 86 percent lower risk of myocardial infarction compared with men with two slow alleles who drank less than weekly. These results suggest that, within the range of moderate drinking, greater exposure time to alcohol (on the basis of more frequent drinking and slower metabolism), may lower one’s risk of myocardial infarction.

The researchers also found that among daily drinkers, “good” HDL-C cholesterol increased with the number of slow ADH3 alleles—that is, daily drinkers with two slow ADH3 alleles had higher HDL-C levels than did daily drinkers with no slow ADH3 allele levels; men with one slow allele had intermediate HDL-C levels. This finding provides a plausible explanation for the gene-related variation in the relationship between alcohol consumption and risk of myocardial infarction described in the study.

Danish investigators reported intriguing findings in a study of 3,383 men (Hein et al. 1993). The investigators compared the risk of cardiovascular mortality among men with different Lewis blood group types. Much like the common ABO blood group system, a person’s Lewis blood type can include just an “a” component (a+b−), just a “b” component (a−b+), both components (a+b+), or neither component (a−b−). People with the a−b− blood type seem to be at higher risk for diabetes and cardiovascular mortality than people with other Lewis blood types. In the study, men with Lewis blood group type a−b− who consumed 22 or more drinks per week had an 80 percent lower risk of coronary heart disease than did men who consumed 0–10 drinks per week. Among men with other Lewis blood group types, however, alcohol consumption was not appreciably related to the risk of heart disease.

Taken together, these two studies suggest that genetic factors that influence potentially beneficial variables linked to alcohol use (e.g., HDL-C levels) or the baseline risk of heart disease (e.g., Lewis blood type groups) may modify the link between alcohol consumption and heart disease in important and informative ways.

## Putting It Together

This article has explored whether alcohol consumption per se is responsible for the lower risk of coronary heart disease among moderate drinkers. Based on the results of the meta-analysis of randomized trials by Rimm and colleagues (1999), the answer appears to be yes. If alcohol consumption indeed influences HDL-C, triglyceride, and fibrinogen levels to the degree documented in the meta-analysis, consumption of two standard drinks daily would be expected to lower a person’s risk of coronary heart disease by nearly 25 percent, a figure that agrees well with the results of observational studies.

Obviously, however, alcohol consumption also has serious and important health effects other than those related to coronary heart disease, which are reviewed elsewhere in this journal issue. Achieving a balance between the health risks and benefits of alcohol consumption remains difficult, as each person has a different susceptibility to the adverse health consequences associated with alcohol consumption. Because each person has a unique combination of factors—such as age, sex, and family history—that influence that person’s risk of specific diseases potentially caused or prevented by alcohol use, the balance of the risks and benefits of alcohol consumption for each person likewise will be unique. Accordingly, a young woman with a family history of alcoholism should weigh the decision of how much alcohol to drink (if any) differently than should a middle-aged man with a family history of premature heart disease.

One approach to examining the combined results of potentially detrimental and beneficial effects associated with alcohol consumption is to assess the overall rates of death in people who consume different amounts of alcohol. Such studies of all-cause mortality can combine the baseline risk of dying from each specific disease with the increase or decrease in the risk for that disease associated with alcohol consumption. Obviously, observational studies of all-cause mortality are susceptible to the same concerns discussed earlier regarding studies of coronary heart disease. Nevertheless, the apparent agreement of clinical and observational studies regarding the relationship between alcohol consumption and coronary heart disease provides reassurance about the validity of these reports.

Given that over 30 percent of deaths in the United States are attributable to heart disease, making it the nation’s leading cause of death, it is not surprising that observational studies show that moderate drinkers have lower overall death rates than do abstainers or heavy drinkers. For example, in an American Cancer Society study of 490,000 adults, death rates among middle-aged and elderly men and women were lowest among people who consumed approximately one drink per day (Thun et al. 1997). In fact, death rates among these moderate drinkers were approximately 20 percent lower than among abstainers. The level of alcohol consumption associated with the lowest overall death rate, however, differed substantially based on the participants’ age and risk of heart disease. For example, among participants aged 30–59 years and free of hypertension, diabetes, or cardiovascular disease, the lowest death rate was found with a consumption of less than 1 drink daily. Conversely, among participants aged 60–79 years and with one of these conditions, the lowest death rate occurred with a consumption of three drinks per day.

In light of the substantial and often contradictory evidence regarding the health effects of alcohol consumption, one cannot make a simple recommendation regarding the “optimal” level of alcohol consumption. In the absence of such a straightforward recommendation, people should consult their physicians regarding the safety or risk of alcohol consumption and make personalized decisions accordingly.

## Footnotes

1 A standard drink is defined as 12 fluid ounces of beer, 5 fluid ounces of wine, or 1.5 fluid ounces of distilled sprits and contains approximately 0.5 ounces (i.e., 14 grams) of alcohol.

2 The term “less welcoming” in this instance refers to the degree of parental warmth and cohesion in a child’s life as measured on a “Childhood Environmental Strengths Scale” administered by trained psychological interviewers.

3 Antioxidants are substances such as vitamin E that can eliminate highly reactive molecules called free radicals, which can damage cells and various cellular components.
